# Actin disruption is associated with altered YAP/TAZ-responsive transcription and reduced migration during palatal shelf elevation

**DOI:** 10.3389/fcell.2026.1962374

**Published:** 2026-10-01

**Authors:** Yang Bai, Jiarong Liu, Siqi Tang, Yuxin Zhang, Chang Liu, Fangchun Chen, Xiaoming Wang, Xiaoxiao Pang

**Affiliations:** 1 Chongqing Key Laboratory of Oral Diseases and Biomedical Sciences, Chongqing Municipal Key Laboratory of Oral Biomedical Engineering of Higher Education, Stomatological Hospital of Chongqing Medical University, Chongqing, China; 2 Chongqing Technology Innovation Center of Smart Dental Medical Devices, Chongqing, China; 3 Key Laboratory of Dental Maxillofacial Reconstruction and Biological Intelligence Manufacturing, School of Stomatology, Lanzhou University, Lanzhou, China; 4 Innovation and Transformation of Dental Medical Devices Engineering Research Center of Chongqing Education Commission of China, Chongqing, China

**Keywords:** actin cytoskeleton, CCN1, CCN2, cell migration, mechanotransduction, palatal development, YAP/TAZ

## Abstract

**Introduction:**

Palatal shelf elevation requires coordinated mesenchymal remodeling, but the relationship between actin organization, mechanosensitive transcription, and mesenchymal motility remains incompletely defined. This study examined how actin disruption relates to YAP/TAZ‐responsive transcription and palatal mesenchymal behavior.

**Methods:**

Primary mouse embryonic palatal mesenchymal cells and E13.5 palate organ cultures were treated with latrunculin A (Lat‐A). Actin organization, YAP localization, wound closure, and palatal shelf orientation were assessed. GA‐017, a LATS1/2 inhibitor that can activate both YAP and TAZ, was used to examine responses in the Lat‐A background. RNA sequencing and reanalysis of public single‐cell multiomic and spatial transcriptomic datasets characterized the associated transcriptional programs.

**Results:**

Lat‐A disrupted filamentous actin, reduced wound closure, and altered YAP localization. Reduced wound closure persisted in the presence of mitomycin. GA‐017 partially opposed the Lat‐A‐associated changes in wound closure and transcription. RNA sequencing identified 674 genes with opposite-direction changes in the Lat‐A‐versus-control and Lat‐A‐plus‐GA‐017‐versus‐Lat‐A comparisons, including Ccn1, Ccn2, Ankrd1, and Amotl2. In organ culture, Lat‐A was associated with reduced F‐actin, YAP, CCN1, and CCN2 signals and abnormal palatal shelf orientation. Public datasets provided complementary evidence for the developmental expression and spatial overlap of YAP/TEAD‐, CCN‐, actin‐, and migration-related programs.

**Discussion:**

These findings link actin disruption to altered LATS‐sensitive YAP/TAZ‐responsive transcription and reduced palatal mesenchymal wound closure during palatal elevation. CCN1 and CCN2 are candidate components of this response.

## 1 Introduction

Perturbation of essential cellular processes during embryogenesis can disrupt morphogenesis without overt cytotoxicity (Knudsen and Kleinstreuer, 2011; Wale and Gardner, 2016). Secondary palatogenesis depends on coordinated growth, elevation, and fusion of bilateral palatal shelves (Bush and Jiang, 2012; Li et al., 2017; Deshpande and Goudy, 2019), among which palatal elevation is especially rapid and requires local mesenchymal remodeling and tissue-generated forces (Deshpande and Goudy, 2019; Goering et al., 2025). However, how mesenchymal cytoskeletal dynamics are coupled to transcriptional programs during this transition remains poorly defined.

Filamentous actin (F-actin) coordinates cell shape, polarity, force generation, and directional migration (Papakonstanti and Stournaras, 2008; Blanchoin et al., 2014), and actomyosin organization has been implicated in palatal shelf elevation, particularly in the middle and posterior regions (Chiquet et al., 2016; Pang et al., 2021; Bai et al., 2023). We previously reported that mesenchymal β-catenin loss delayed elevation and reduced F-actin assembly (Pang et al., 2021), whereas mesenchymal-specific inactivation or overexpression of β-catenin resulted in decreased or increased α-actinin-4 expression, which is important for F-actin cross-linking (Wang et al., 2023). These observations implicate actin organization in shelf remodeling but do not establish how actin polymerization affects palatal mesenchymal behavior.

Yes-associated protein (YAP) and transcriptional coactivator with PDZ-binding motif (TAZ) are mechanosensitive transcriptional coactivators that connect cytoskeletal state to proliferation, survival, migration, and differentiation (Varelas, 2014; Wu and Guan, 2021; Engel-Pizcueta and Pujades, 2021; Panciera et al., 2017; Halder et al., 2012; Meli et al., 2023). Mesenchymal deletion of YAP/TAZ delayed palatal shelf elevation and caused cleft palate in mice (Goodwin et al., 2020). Previous studies have linked F-actin integrity and mechanical cues to YAP nuclear localization (Das et al., 2016; Nakamura, 2024; Lin et al., 2025), including an F-actin-dependent mechanism in cells lacking cell-cell contact (Das et al., 2016). We therefore hypothesized that disruption of F-actin organization would be accompanied by altered YAP/TAZ-responsive transcription and reduced palatal mesenchymal motility.

To test this hypothesis, we used latrunculin A (Lat-A) as a reversible actin-disrupting probe in primary MEMP cells and E13.5 palate organ cultures. We examined wound closure, YAP localization, transcriptomic responses to GA-017 in the Lat-A background, and shelf orientation in organ culture. Because GA-017 inhibits LATS1/2 and can activate both YAP and TAZ, these pharmacological experiments were interpreted as evidence for a LATS-sensitive YAP/TAZ-responsive program rather than a YAP-specific rescue. Independent single-cell multiomic and spatial transcriptomic datasets were reanalyzed to determine whether the implicated programs occurred in the relevant developmental compartment.

## 2 Materials and methods

### 2.1 Animals

Eight-week-old C57BL/6J mice were purchased from Ensiweier Bio Inc (Chongqing, China). Timed embryos were collected at E12.5, E13.5, E14.0, or E14.5, with three fetuses from each dam allocated at each stage. All procedures were approved by the Ethics Committee of the Stomatological Hospital of Chongqing Medical University (CQHS-REC-2025 [LSNo.24]).

### 2.2 Isolation and culture of MEMP cells

Palatal shelves were microdissected from E13.5 embryos at the mandibular junction. Tissues were incubated in Dispase II (2.4 U/mL in phosphate-buffered saline) at 4 °C for 18 h, washed three times, and incubated with 0.25% trypsin-EDTA at 37 °C for 15 min. Digestion was stopped with DMEM/F12 containing 10% fetal bovine serum (FBS). The suspension was passed through a 40-µm mesh and centrifuged at 1,000 rpm for 5 min. Cells were resuspended in DMEM/F12 containing 10% FBS and plated. Passage three cells were used for all functional assays.

### 2.3 Immunofluorescence staining and quantification

Cells were treated with 1 µM Lat-A (MCE, HY-16929) or 1 µM Lat-A plus 10 µM GA-017 (Solarbio, IYT0805) for 1 h or with 1 µM cytochalasin D (MCE, HY-N6682) for 1 or 6 h, with DMSO serving as the vehicle control. Following fixation, permeabilization, and blocking, cells were incubated with a primary antibody, followed by a fluorescent secondary antibody. F-actin and nuclei were labelled with a phalloidin-based F-actin staining kit and DAPI, respectively. Antibody manufacturers and dilutions are listed in Supplementary Table S1.

For quantification of the YAP cytoplasmic-to-nuclear ratio under Lat-A treatment, nuclear regions were segmented using DAPI staining, and surrounding perinuclear regions, excluding all nuclear pixels, were used as cytoplasmic proxies. The YAP C/N ratio was calculated by dividing the mean background-corrected perinuclear fluorescence intensity by the mean nuclear intensity. Up to 10 or 20 cells from three biological replicates per group were randomly selected after quality control. This analysis provides a semi-quantitative estimate of YAP localization rather than a whole-cytoplasmic measurement.

For quantification of the YAP cytoplasmic-to-nuclear ratio under cytochalasin D treatment, nuclear regions were defined by DAPI, and visible cytoplasmic regions were estimated from F-actin staining, excluding all nuclear masks and a one-pixel margin. Cells with ambiguous boundaries, unstable segmentation, or excessive near-saturation were excluded using consistent quality-control criteria across groups. The cytoplasmic-to-nuclear (C/N) ratio was calculated as the mean cytoplasmic YAP intensity divided by the mean nuclear YAP intensity. Cell-level ratios were averaged within each field, with one field representing each of five independent biological replicates per group.

Fluorescence images were quantified using custom Python scripts. For tissue sections, three sections from three biological replicates per group were quantified, and tissue regions were defined from the DAPI channel independently of the target fluorescence signal. Mean target fluorescence intensity within these regions was calculated after subtraction of the median intensity in the surrounding background. For cultured cells, five fields of view were analyzed per group, with one field from each of five independent biological replicates. Background-corrected integrated CCN1 or CCN2 fluorescence within each field was multiplied by the calibrated pixel area and divided by the number of DAPI-positive nuclei in the same field. Scale bars and image annotations were excluded from the measurements. Consistent analysis parameters were applied across groups.

### 2.4 Histology and fluorescence staining

Palatal tissues were fixed overnight in 4% paraformaldehyde (PFA), dehydrated through graded ethanol, cleared in xylene, embedded in paraffin, sectioned at 4 μm, and stained with hematoxylin and eosin. For immunofluorescence, deparaffinized sections underwent heat-induced antigen retrieval in 10 mM sodium citrate (pH 6.0). MEMP cells were fixed in 4% PFA, permeabilized with 0.5% Triton X-100, and blocked with 5% goat serum. Tissue sections and cells were incubated with primary antibodies overnight at 4 °C and fluorophore-conjugated secondary antibodies for 1 h at room temperature. Nuclei were counterstained with 4′,6-diamidino-2-phenylindole, and images were acquired with an Olympus fluorescence microscope. The phalloidin-based F-actin staining kit was used for cultured MEMP cells and paraffin sections, whereas Actin-Tracker Red (Beyotime, C22055) was used for organ cryosections. Antibody manufacturers and dilutions are listed in Supplementary Table S1.

### 2.5 Reverse transcription-quantitative PCR

RNA was isolated using the RNAeasy Plus Animal RNA Isolation Kit (Beyotime). cDNA was generated with the ABScript II cDNA First-Strand Synthesis Kit and analyzed using ABScript II SYBR Green chemistry (ABclonal). Relative expression was normalized to Gapdh. Primer sequences are provided in Supplementary Table S2.

### 2.6 Wound closure and cell viability assays

Passage 3 MEMP cells were seeded in ibidi culture inserts in six-well plates and serum-starved for 16 h. After starvation, cells were maintained in DMEM/F12 containing 3% FBS and exposed to the indicated combinations of untreated medium, DMSO vehicle (0.5 μL/mL, MCE, HY-Y0320), 1 µM Lat-A, 10 μM GA-017, and 1.5 µM mitomycin (MCE, HY-13316). Inserts were removed to create standardized cell-free gaps, debris was removed by three PBS washes, and the same fields were imaged at 0, 12, and 24 h. Because gap closure can reflect both migration and changes in cell number, it is referred to as wound closure unless proliferation and survival were assessed independently. For CCK-8 assays, cells were exposed to Lat-A at 0.5, 1.0, or 1.5 µM or to mitomycin at 0.5, 1.0, 1.5, or 2.0 µM. Absorbance at 450 nm was measured after 2 h with CCK-8 reagent.

Wound closure was quantified from the residual wound area at 0, 12, and 24 h. Cell-front boundaries were delineated and visually verified to exclude cellular regions from the wound area. Closure was calculated as [1-residual area/mean initial area of the corresponding group] × 100%. Data represent mean ± SD from three independent biological replicates per group. Groups were compared at each time point using two-sided, unpaired Welch’s t-tests, with P values reported.

### 2.7 RNA sequencing and bioinformatic analysis

Total RNA from control, Lat-A, and Lat-A plus GA-017 MEMP cells was isolated with TRIzol. RNA quality and concentration were assessed with an Agilent 5300 Bioanalyzer and NanoDrop ND-2000. Libraries were prepared from 1 µg RNA using the Illumina Stranded mRNA Prep Ligation kit. Reads were filtered with fastp, aligned with HISAT2, assembled with StringTie, and quantified with RSEM. Differential expression was tested with DESeq2. Genes with an absolute log2 fold change ≥1 and false-discovery rate (FDR) < 0.05 were considered significant. Opposite-direction genes were significant in both the Lat-A-versus-control and Lat-A-plus-GA-017-versus-Lat-A contrasts and changed in opposing directions. Gene Ontology (GO) biological process enrichment analysis was performed in R using annotations from org.Mm.e.g.,.db and GO.db. Genes showing significant opposite-direction changes in both contrasts were tested against the background of GO-mapped genes retained in the RNA-seq analysis using a hypergeometric test. P values were adjusted using the Benjamini–Hochberg method, and terms with an adjusted P value <0.05 were considered significantly enriched.

### 2.8 *Ex vivo* palate organ culture

Palatal organs dissected from E13.5 embryos were cultured on stainless-steel meshes in DMEM/F12 containing 10% FBS, 1% penicillin-streptomycin, and 1% glutamine. Cultures received DMSO vehicle or 10 µM Lat-A for 48 h, with medium replacement every 24 h. Samples were fixed in 4% PFA and processed for histology or cryoprotection in 15% and 30% sucrose. Cryoprotected samples were embedded in OCT compound and sectioned at 8 µm. Sections were stained for 1 h with Actin-Tracker Red (Beyotime, C22055; 1:100) and counterstained with DAPI.

### 2.9 Public dataset reanalysis

Processed single-cell multiomic data from developing mouse secondary palate were obtained from GEO accession GSE218576 (Yan et al., 2024). Mesenchyme-like cells were identified with established markers. RNA counts and prespecified YAP/TEA domain transcription factor (TEAD)-target, actin-regulation, and migration/adhesion scores were aggregated by public sample/library. Paired scATAC-seq matrices were used to assess positively linked peaks near Ccn1, Ccn2 and TEAD-motif-associated accessibility.

Four E13.5 sections from the mouse organogenesis spatiotemporal transcriptomic atlas (MOSTA) (Chen et al., 2022) were analyzed with the supplied expression matrices, spatial coordinates, and annotations. A marker-refined oral/craniofacial region was defined computationally. Spatial co-expression was evaluated separately in each section using Spearman correlation. Cells and spatial bins were not treated as independent biological replicates. Full analytical details are provided in the Supplementary Methods.

### 2.10 Statistical analysis

Statistical analyses were performed in GraphPad Prism 8.0. Two-group comparisons used two-sided Student's t-tests or Welch’s t-tests, as indicated. One-way or two-way analysis of variance was used for multigroup designs. P < 0.05 was considered statistically significant. Data are presented as mean ± standard deviation unless stated otherwise. The biological replicate unit was the independent cell experiment, embryo/organ, or public sample/library, as appropriate.

## 3 Results

### 3.1 Regional cell density and F-actin organization change during palatal shelf elevation

Histological examination showed the expected medial mesenchymal bulge in the posterior palatal shelves during elevation (Figure 1A). DAPI-based quantification showed lower cell density on the medial than on the lateral side of the posterior palatal mesenchyme before, during, and after elevation, whereas the corresponding medial-lateral comparison was not significant in the anterior shelves (Figure 1B). β-catenin staining was also weaker in the sparse medial region than in the surrounding condensed mesenchyme (Supplementary Figure S1), indicating an association between regional cell density and cell-cell junction-related staining. Phalloidin staining further revealed localized F-actin enrichment at the upper aspect of the medial bulge at E14.0 (Figure 1C). Together, these results suggest that regional changes in cell packing and F-actin organization accompanied posterior shelf elevation.

**FIGURE 1:**
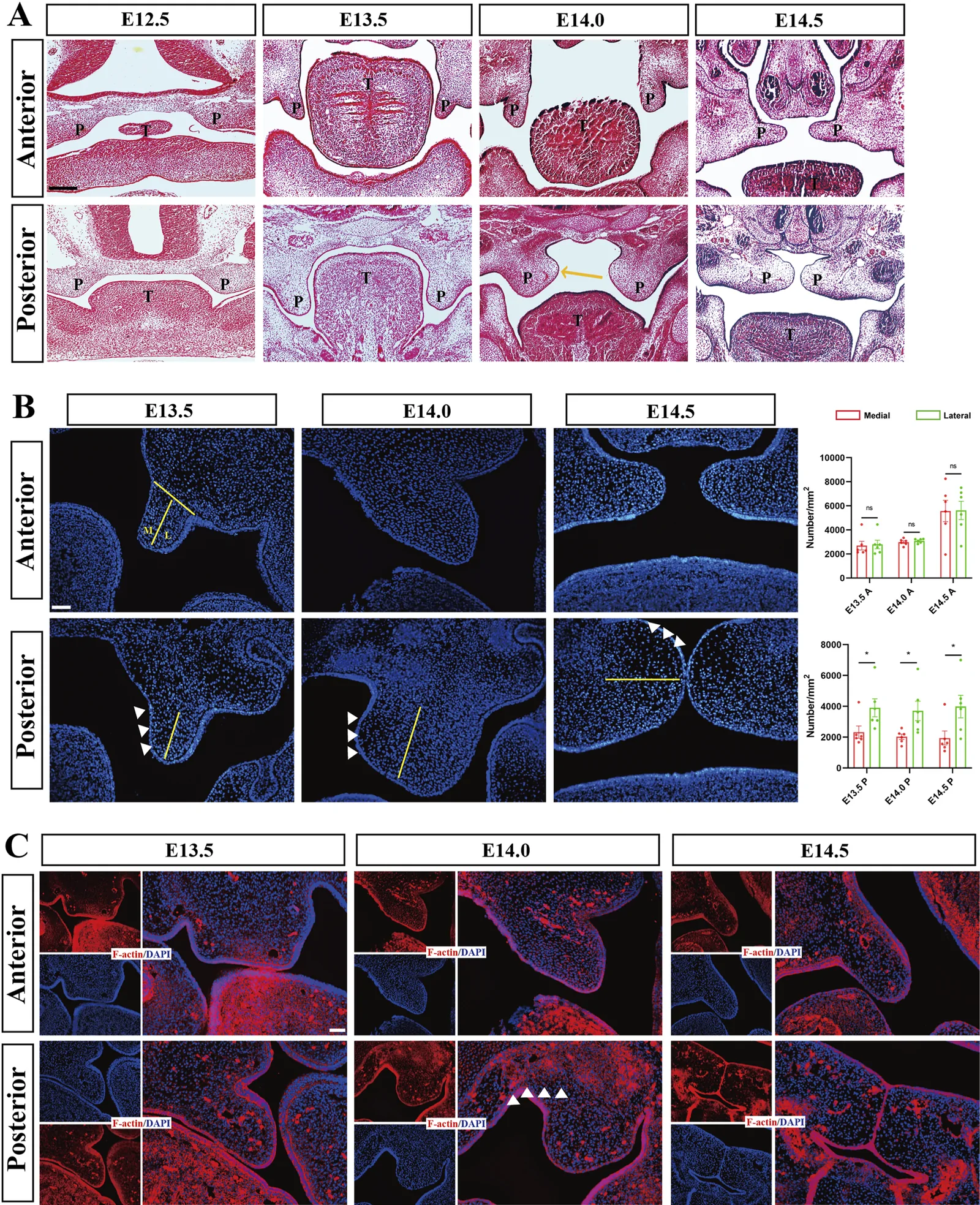
Regional cell density and F-actin organization during palatal shelf elevation. **(A)** Hematoxylin and eosin staining showed a medial bulge in the posterior palatal shelves. P, palatal shelf; T, tongue. **(B)** DAPI staining was used to compare medial (M) and lateral (L) cell density in anterior and posterior shelves. The medial-lateral difference in the anterior shelf was not significant (ns) (n = 6). **(C)** F-actin was enriched at the upper aspect of the posterior mesenchymal bulge at E14.0. The yellow arrow indicates the bulge; white arrows in B indicate the lower-density medial region; white arrows in C indicate increased F-actin staining. Data are mean ± SEM. Scale bar, 200 µm **(A)**, 50 µm **(B,C)**. *P < 0.05; ns, not significant.

### 3.2 Lat-A disrupts F-actin organization and reduces MEMP cell wound closure

Primary MEMP cells isolated from E13.5 palatal shelves displayed a spindle-shaped morphology and little E-cadherin expression, consistent with mesenchymal enrichment (Supplementary Figure S2). Dose-response experiments with Lat-A (0.1–2.0 µM) revealed progressive disassembly of the peripheral F-actin network, accompanied by the appearance of phalloidin-positive puncta (Supplementary Figure S3A). The effects of 1 µM Lat-A were largely reversible after drug washout, with normal cell morphology and F-actin organization restored within 24 h (Supplementary Figure S3B). We therefore used 1 µM Lat-A as a reversible actin-disrupting condition in subsequent cell experiments.

The wound-healing assay showed that Lat-A exposure reduced wound closure and left a persistent cell-free gap after 24 h relative to the vehicle control (Figure 2A). Because Lat-A also reduced the CCK-8 signal at 1.0 and 1.5 µM (Supplementary Figure S4A), changes in cell number, survival, or metabolic activity could contribute to this phenotype. Under treatment with 1.5 μM mitomycin C, the CCK-8 signal showed no apparent increase between 12 and 24 h (Supplementary Figure S4B). This concentration was used in subsequent wound-closure assays to limit the contribution of proliferation. The results showed that Lat-A remained associated with significantly reduced wound closure at 24 h (Supplementary Figure S4C). This observation suggests that Lat-A-mediated F-actin disruption may directly affect MEMP cell motility.

**FIGURE 2:**
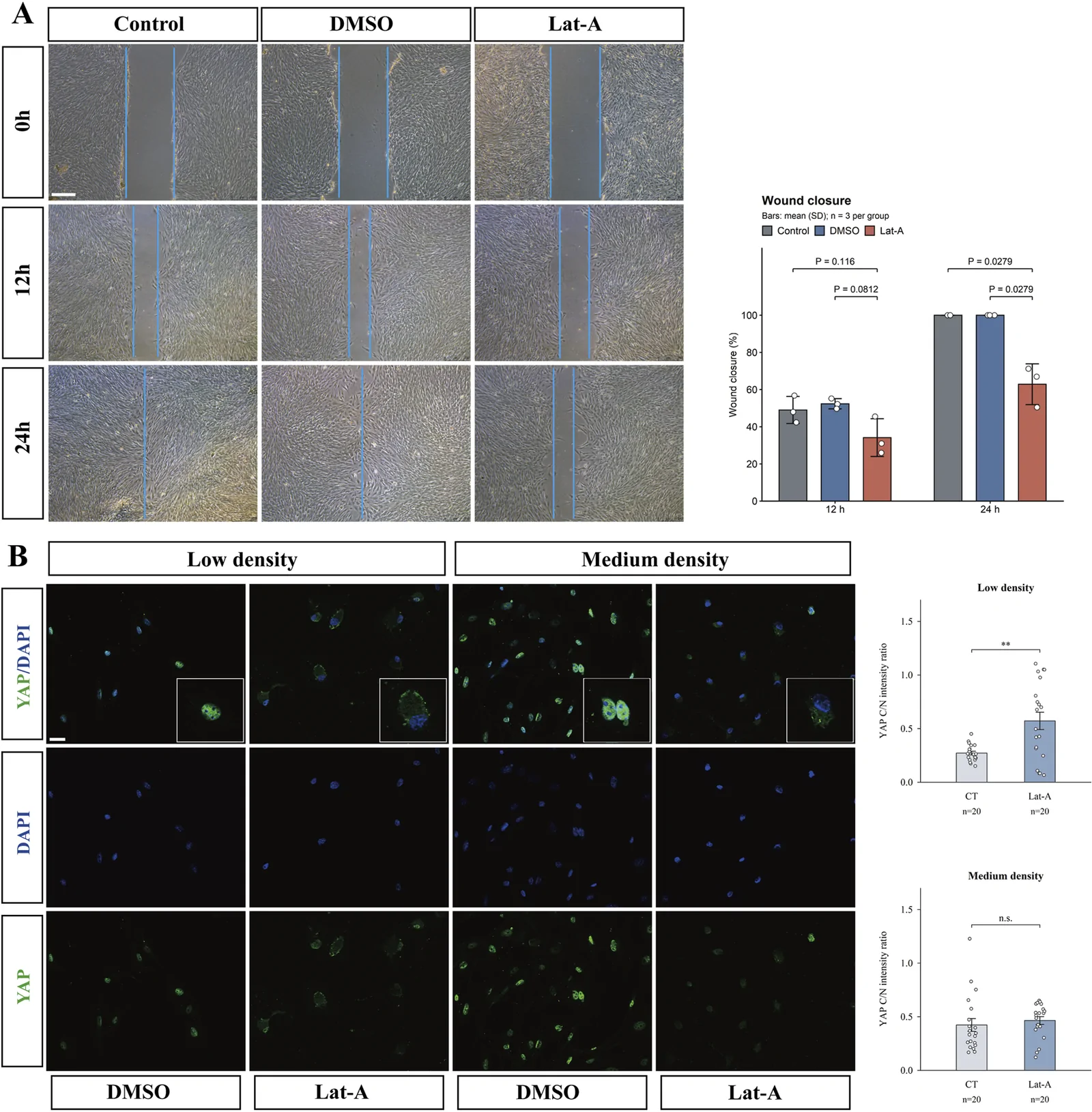
MEMP cell wound closure and YAP localization after Lat-A-mediated F-actin disruption. **(A)** Representative wound fields at 0, 12, and 24 h. Untreated control, DMSO vehicle, and 1 µM Lat-A conditions are indicated. Blue lines delineate the cell-free gap. Quantitative wound closure analysis shows a significantly reduced wound closure ratio with Lat-A treatment at 24 h. **(B)** Representative YAP and DAPI fluorescence images at low and medium cell densities. Quantification (20 segmented cells randomly selected from three biological replicates per condition) showed a Lat-A-associated difference at low density but not at medium density. Scale bar, 250 µm **(A)**, 50 µm **(B)**. **P < 0.01; ns, not significant.

### 3.3 F-actin disruption redistributes YAP away from the nucleus in MEMP cells

Since YAP localization is sensitive to cell density and cytoskeletal tension, we first examined untreated MEMP cells across a density series (5,000, 10,000, and 200,000 cells/mL). The cytoplasmic-to-nuclear YAP fluorescence ratio differed among densities (n = 10 segmented cells per condition randomly selected from three biological replicates; one-way ANOVA, P = 0.00174; Supplementary Figure S5). Low- and medium-density cultures were then compared after Lat-A exposure (Figure 2B). Two-way ANOVA showed a treatment effect (P = 0.00229) and a density-by-treatment interaction (P = 0.01929; Figure 2B). Prespecified within-density comparisons showed a difference between vehicle and Lat-A at low density (Holm-adjusted P = 0.00317), but not at medium density (P = 0.558).

However, because Lat-A treatment disrupted F-actin staining and made it difficult to define cell boundaries precisely (Figure 3A), we could not reliably delineate the entire cytoplasm for YAP quantification under this condition. To address this, we used cytochalasin D, another F-actin-disrupting agent that still allowed clear cell boundary identification. As expected, cytochalasin D treatment also reduced nuclear YAP enrichment, resulting in a higher cytoplasmic-to-nuclear ratio (Supplementary Figure S6). Together, these data suggest that intact F-actin organization is associated with the maintenance of nuclear YAP in sparsely plated MEMP cells.

**FIGURE 3:**
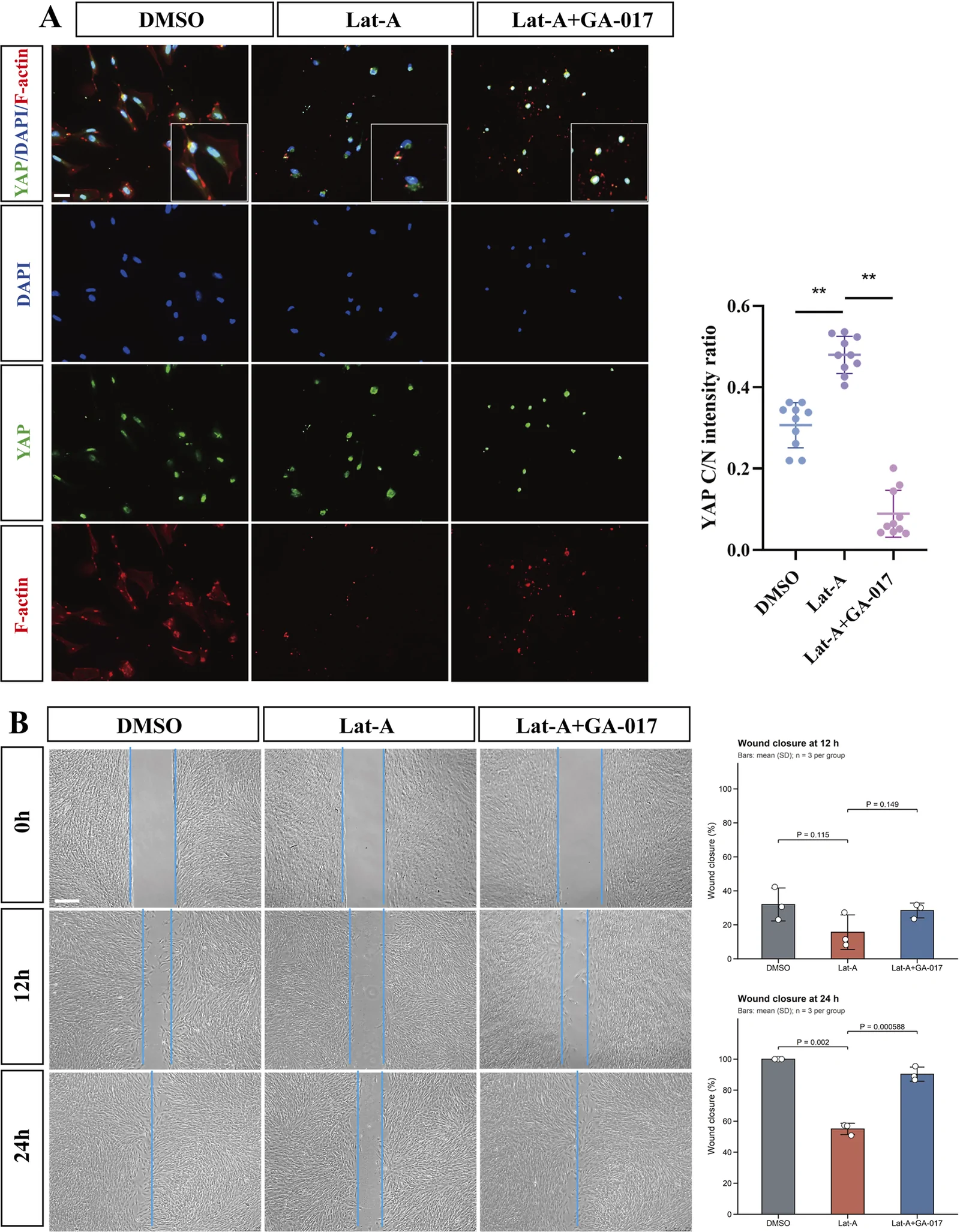
GA-017 opposes Lat-A-associated changes in YAP localization and wound closure. **(A)** Representative F-actin (red), YAP (green), and DAPI (blue) images after treatment with DMSO vehicle, 1 µM Lat-A, or 1 µM Lat-A plus 10 µM GA-017. Quantification (10 segmented cells randomly selected from three biological replicates per condition) showed significantly decreased cytoplasmic-to-nuclear YAP fluorescence ratios in the 1 µM Lat-A plus 10 µM GA-017 group. **(B)** Representative wound fields at 0, 12, and 24 h under the same treatment conditions. Quantification showed a significantly increased wound closure ratio with 1 µM Lat-A plus 10 µM GA-017 treatment at 24 h. Blue lines delineate the cell-free gap. Scale bar, 50 µm **(A)**, 250 µm **(B)**. **P < 0.01.

### 3.4 GA-017 opposes Lat-A-associated changes in YAP localization and wound closure

We next used 10 μM GA-017, an ATP-competitive LATS1/2 inhibitor, in the Lat-A background. The fluorescence panels included F-actin, YAP, and DAPI channels and showed predominantly nuclear YAP signal in the Lat-A plus GA-017 condition, whereas Lat-A alone was associated with a more cytoplasmic pattern (Figure 3A). GA-017 also significantly reduced the persistent cell-free gap observed after 24 h of Lat-A exposure (Figure 3B). Since LATS1/2 inhibition can activate both YAP and TAZ to affect proliferation and survival, these observations support involvement of a LATS-sensitive YAP/TAZ program in this process.

### 3.5 GA-017 opposes a subset of Lat-A-associated transcriptional changes in MEMP cells

To characterize transcriptional changes associated with Lat-A treatment and GA-017 co-treatment, we performed RNA sequencing of control, Lat-A-treated, and Lat-A plus GA-017-treated MEMP cells. Principal-component analysis separated samples by treatment condition and showed high within-condition similarity (Figure 4A). Lat-A altered 2,404 genes relative to control, whereas GA-017 altered 931 genes in the Lat-A background. Effect sizes for the two contrasts were inversely correlated across the transcriptome (Spearman’s ρ = −0.684; Figure 4B). A total of 674 genes were significant in both contrasts and changed in opposite directions. This opposite-direction set included Ccn1, Ccn2, Ankrd1, and Amotl2, which are responsive to YAP/TAZ-TEAD signaling in other contexts (Figures 4C,D).

**FIGURE 4:**
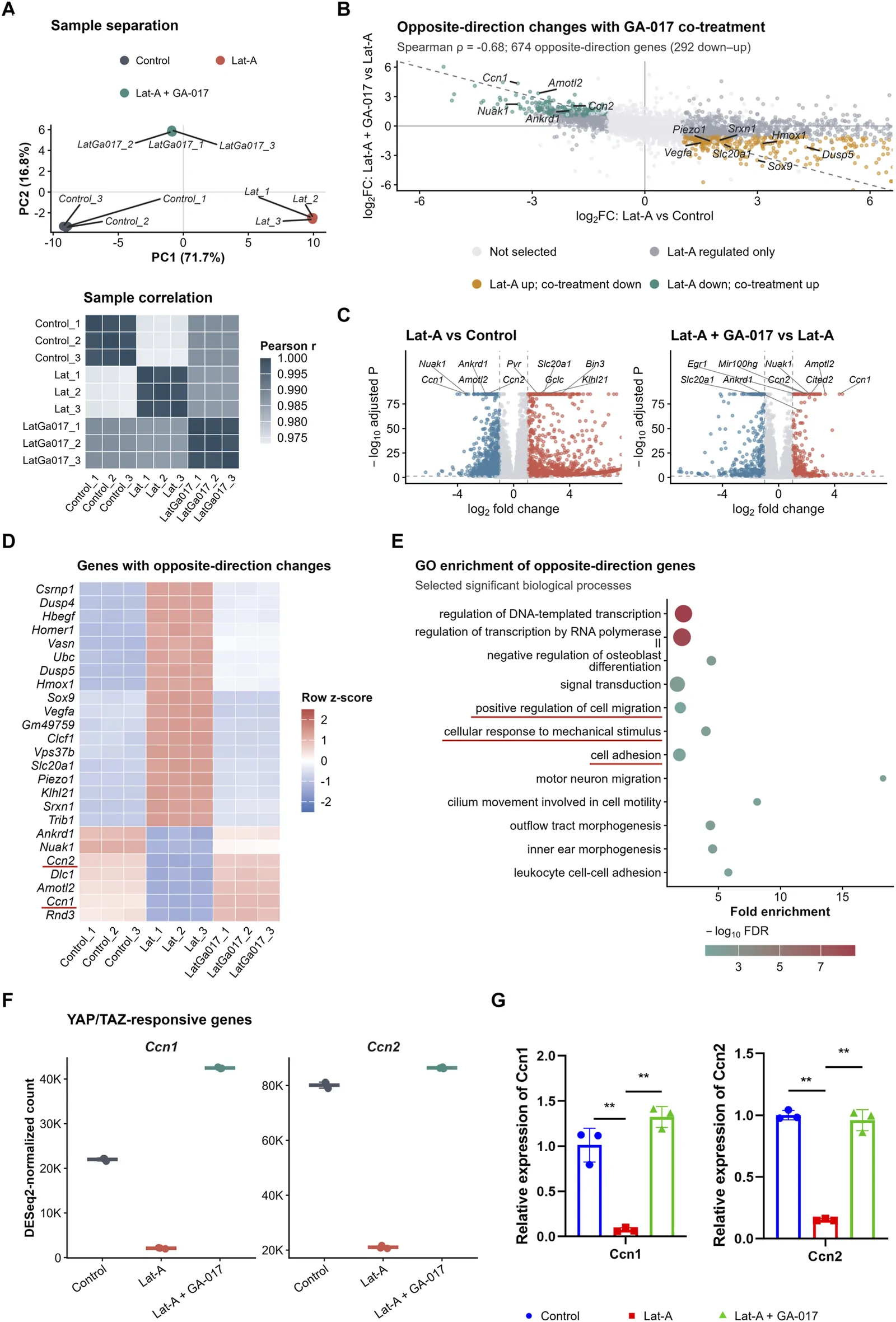
GA-017 opposes part of the Lat-A-associated transcriptional response. **(A)** Principal-component analysis (PCA) and Pearson correlation heatmap of variance-stabilized expression values across samples. **(B)** Differential expression for Lat-A versus control and Lat-A plus GA-017 versus Lat-A. Each point represents a gene retained after expression filtering. Genes significant in both contrasts (FDR <0.05 and absolute log2 fold change ≥1) and changing in opposite directions are highlighted; the dashed line indicates equal and opposite effect sizes. **(C)** Volcano plots for both contrasts; red denotes upregulated genes and blue denotes downregulated genes. **(D)** Row z-scores of variance-stabilized expression for representative opposite-direction genes. **(E)** Selected Gene Ontology biological process terms enriched among the opposite-direction genes. **(F)** DESeq2-normalized counts for Ccn1 and Ccn2. **(G)** RT-qPCR analysis of Ccn1 and Ccn2 expression in the three groups. Data are mean ± SD. **P < 0.01.

Additionally, the opposite-direction gene set was enriched for responses to mechanical stimuli, cell adhesion, positive regulation of cell migration, and related motility processes (Figure 4E). DESeq2-normalized counts showed suppression of Ccn1 and Ccn2 after Lat-A exposure and higher expression after addition of GA-017 (Figure 4F); RT-qPCR reproduced these directional changes (Figure 4G). These results suggest that GA-017 opposes a subset of Lat-A-associated expression changes in the Lat-A background, including canonical YAP/TAZ-responsive genes.

### 3.6 Lat-A impairs palatal shelf elevation in *ex vivo* organ culture

We next examined whether Lat-A exposure was accompanied by a tissue-level morphogenetic phenotype. We collected 35 embryos from 3 litters, and E13.5 palate organs were cultured for 48 h with DMSO vehicle (17 embryos) or 10 µM Lat-A (18 embryos). The results showed that 14/17 palatal shelves elevated and fused in the DMSO-treated group, while 2/18 palatal shelves elevated and fused in the Lat-A group. Representative control shelves adopted an elevated orientation, whereas Lat-A-treated shelves retained an abnormal orientation (Figures 5A,B). Moreover, significantly lower F-actin, YAP, CCN1, and CCN2 fluorescence signals were observed in the Lat-A condition (Figures 5C–N), and cultured MEMP cells also showed significantly lower CCN1 and CCN2 signals (Figures 5O–T). These observations are consistent with a tissue-level association among actin disruption, altered YAP/TAZ-responsive markers, and abnormal shelf growth.

**FIGURE 5:**
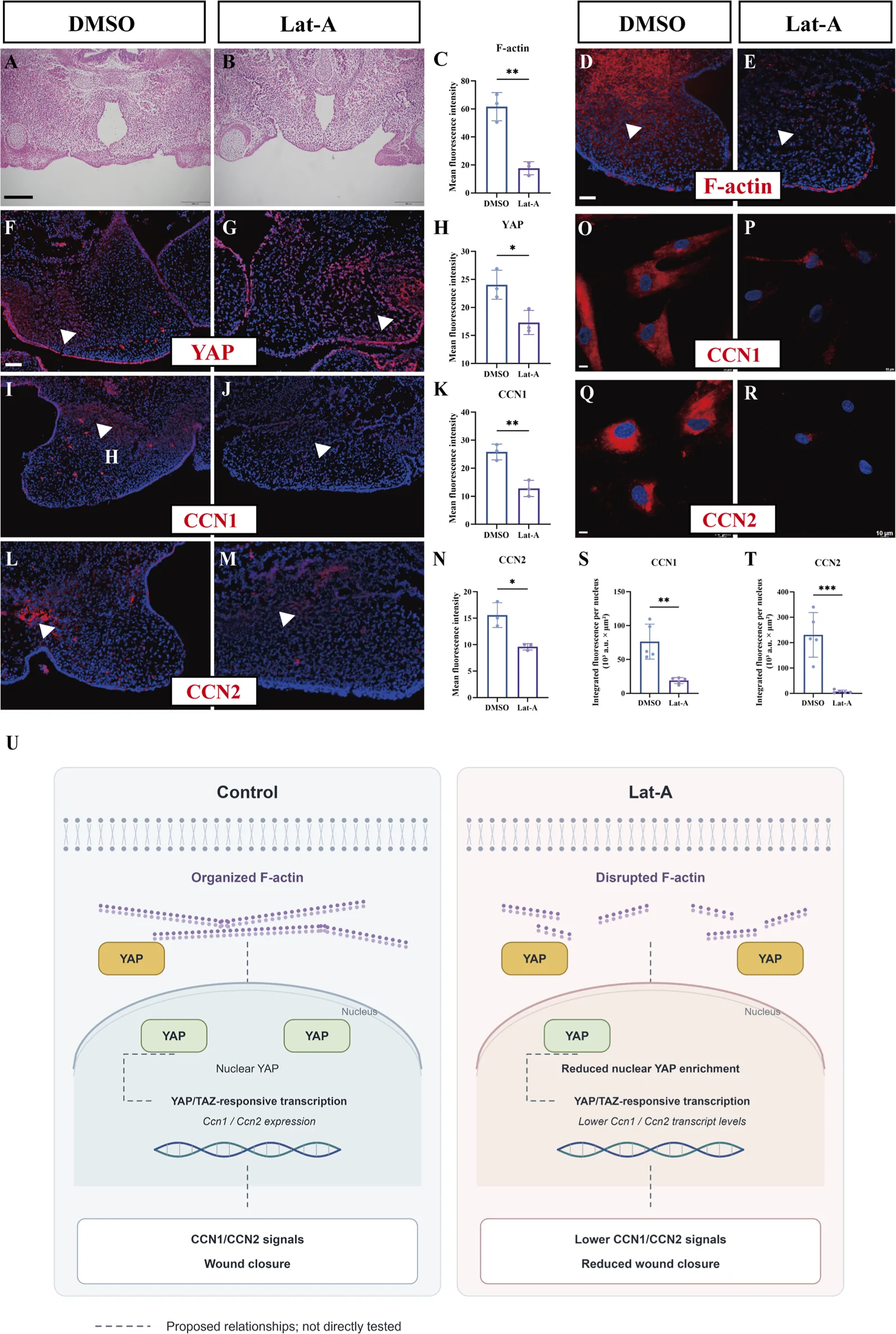
Representative organ-culture and fluorescence observations after Lat-A exposure. **(A,B)** DMSO vehicle and 10 µM Lat-A organ cultures. Hematoxylin and eosin staining showed delayed palatal elevation in the Lat-A treatment group. P, palatal shelf. **(C–E)** F-actin staining and fluorescence quantification in palatal mesenchyme. **(F–H)** YAP staining and fluorescence quantification in palatal mesenchyme. **(I–K)** CCN1 staining and fluorescence quantification in palatal mesenchyme. **(L–N)** CCN2 staining and fluorescence quantification in palatal mesenchyme. **(O–T)** CCN1 and CCN2 staining, and their respective fluorescence quantification in cultured MEMP cells. **(U)** Schematic summary of the observed changes in F-actin organization, YAP localization, YAP/TAZ-responsive transcription, CCN1/CCN2 signals, and wound closure following Lat-A treatment. Dashed connectors indicate proposed mechanistic relationships that were not directly tested. Scale bars: 200 µm **(A,B)**; 50 µm (D, E, F, G, I, J, L, M); and 10 µm (O, P, Q, R). White arrows indicate the displayed F-actin, YAP, CCN1, or CCN2 signals. Data are mean ± SD. *P < 0.05. **P < 0.01. ***P < 0.001.

### 3.7 Independent developmental datasets contextualize YAP/TEAD, CCN, and actin programs in palatal mesenchyme

To assess whether the experimentally observed gene programs were present in an independent developmental context, we reanalyzed the public single-cell multiome dataset GSE218576. Data-driven palatal mesenchymal program states showed coordinated expression of Ccn1, Ccn2, Yap1, Wwtr1, Tead1, Tead4, and actin-regulatory genes (Actb, Actg1, Actn1, Actn4, Cfl1, Arpc2, Arpc3, Cdc42, Rac1, Rhoa, Rock1, and Rock2) (Figure 6A). Expression varied across YAP/TEAD-core, migratory/adhesive, proliferative, matrix-rich, and osteogenic states. The network therefore occupied a heterogeneous mesenchymal compartment rather than a single uniform cell state.

**FIGURE 6:**
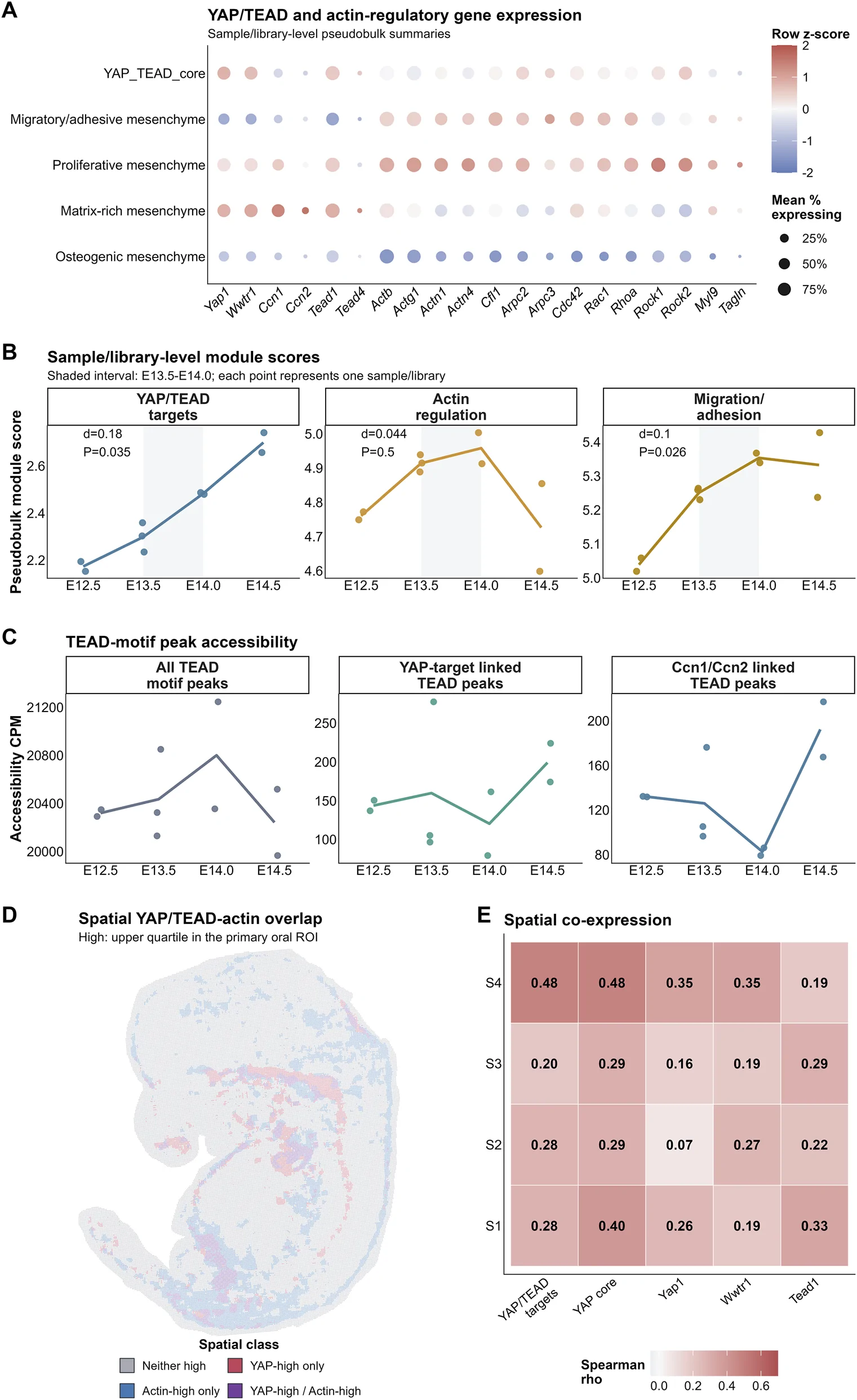
Public single-cell multiomic and spatial data place actin- and YAP/TEAD-related programs in developing palatal mesenchyme. **(A)** Sample/library-level pseudobulk expression of YAP/TEAD-axis, Ccn, and actin-regulatory genes across data-driven palatal mesenchymal program subclusters in GSE218576. Dot color indicates row-scaled mean expression, and dot size indicates the mean fraction of expressing cells. **(B)** Sample/library-level pseudobulk scores for YAP/TEAD-target, actin-regulation, and migration/adhesion gene sets in palatal mesenchyme-like cells from E12.5 to E14.5. The shaded region denotes the E13.5-E14.0 comparison window. Each point represents one public sample/library, and lines connect stage means. Two-sided Welch’s t-tests compared E13.5 (n = 3 libraries) with E14.0 (n = 2 libraries) for the prespecified modules. **(C)** TEAD-motif-associated accessibility from the scATAC-seq peak-count matrix, summarized per sample/library, for all TEAD-motif-containing peaks, peaks linked to YAP-target genes, and peaks linked to Ccn1 or Ccn2 by positive peak-to-gene associations. **(D)** Bivariate spatial map of an E13.5 MOSTA section showing neighborhood-smoothed YAP/TEAD-target and actin-regulation scores. High values were defined as the upper quartile within the primary oral region of interest (ROI). **(E)** Section-level consistency across four E13.5 MOSTA sections. Values represent Spearman correlations between the neighborhood-smoothed actin-regulation score and the indicated YAP/TEAD-related score within the marker-refined candidate ROI. The candidate ROI is a computational, marker-refined approximation of the oral/craniofacial region.

Then, we treated each public sample/library as the aggregation unit. The sample/library-level YAP/TEAD-target score increased by 0.184 units between E13.5 (n = 3 libraries) and E14.0 (n = 2 libraries; Welch’s t-test, P = 0.035). The migration/adhesion score increased by 0.102 units (P = 0.026), whereas the actin-regulation score changed by 0.044 units (P = 0.503) (Figure 6B). Given the small number of public libraries, these comparisons were exploratory and suggest stage-associated increases in YAP/TEAD-target and migratory programs during the E13.5-E14.0 elevation window.

Targeted analysis of the paired scATAC-seq peak-count matrix identified accessible peaks positively correlated with expression of YAP/TAZ-responsive genes, including Ccn1 and Ccn2 (Figure 6C). TEAD-motif-containing accessibility across the Ccn1/Ccn2-linked peak set varied among samples and was higher at E14.5 after lower values at E14.0. This motif-occurrence analysis supports the presence of accessible candidate cis-regulatory regions near the Ccn loci.

Additionally, the E13.5 MOSTA atlas provided an independent spatial assessment. Smoothed YAP/TEAD-target and actin-regulation scores colocalized within discrete regions of the primary oral field (Figure 6D). Across four sections, the actin-regulation score correlated positively with the YAP/TEAD-target score (Spearman’s ρ = 0.20–0.48) and the YAP/TAZ/TEAD-core score (ρ = 0.29–0.48) within a marker-refined oral/craniofacial region of interest (Figure 6E), suggesting YAP/TEAD-related and actin-regulatory transcriptional signatures occupied overlapping spatial domains.

Together with the lower actin-isoform expression at E14.5 than at E13.5 (Supplementary Figure S7), the E14.5 accessibility pattern suggests that actin-transcript dynamics and YAP/TEAD-related regulatory accessibility may become partially decoupled after E14.0.

## 4 Discussion

This study identifies coordinated associations among actin disruption, YAP/TAZ-responsive transcription, and reduced wound closure in palatal mesenchymal cells during the period of shelf elevation. Reversible F-actin disruption was accompanied by altered YAP localization and suppression of a transcriptional program that included Ccn1 and Ccn2. In the Lat-A background, LATS1/2 inhibition with GA-017 opposed part of the wound-closure and transcriptomic response. Although these pharmacological data do not distinguish YAP from TAZ, establish TEAD dependence, or demonstrate migration-specific rescue, they support a functional connection between cytoskeletal organization and YAP/TAZ-responsive transcription. Moreover, the regional tissue pattern is relevant to this interpretation. During elevation, the posterior shelf contained a relatively cell-sparse medial region adjacent to a localized F-actin-rich domain, whereas the same medial-lateral difference was not evident in the anterior shelf. Elevation requires regionally coordinated changes in cell packing, extracellular matrix, and tissue mechanics (Bush and Jiang, 2012; Li et al., 2017; Deshpande and Goudy, 2019; Goering et al., 2025; Chiquet et al., 2016). Weaker β-catenin staining in the sparse medial region agrees with evidence linking mesenchymal β-catenin activity to actin polymerization and elevation (Pang et al., 2021; Wang et al., 2023). These data place cytoskeletal remodeling at a specific anatomical site, although its mechanical role remains unresolved.

Lat-A produced a concentration-dependent loss of filamentous actin, and the cytoskeleton recovered after washout. Reduced CCK-8 signals nevertheless indicate that cell number, survival, or metabolism could contribute to reduced wound closure. Persistence of the phenotype during mitomycin treatment argues against proliferation as the sole explanation, consistent with an effect of Lat-A-induced F-actin disruption on cell motility, but toxicity or metabolic effects need to be further confirmed. Stage- and isoform-specific actin expression in the developing palate (Supplementary Figure S7) further indicates that cytoskeletal remodeling is not a uniform developmental process.

YAP/TAZ provides a plausible route by which cytoskeletal perturbation may alter cell behavior beyond its immediate mechanical effects. The existing cell-level analysis showed a Lat-A-associated localization difference at low density but not at medium density, emphasizing that the response depends on culture context. Additionally, GA-017 opposed the Lat-A-associated YAP localization pattern and reduced the persistent wound gap. Since GA-017 inhibits LATS1/2 and can activate both YAP and TAZ, the data suggest a LATS-sensitive YAP/TAZ-responsive program in this process. Because Yap1, Wwtr1, and TEAD factors were not perturbed and YAP phosphorylation was not measured, genetic epistasis and TEAD-dependent transcriptional assays will be needed to establish the proposed pathway in the future.

The transcriptomic response extends this association to downstream gene regulation. Effect sizes were inversely related between the Lat-A-versus-control and Lat-A-plus-GA-017-versus-Lat-A contrasts, and 674 genes changed significantly in opposite directions. Enrichment for mechanical-stimulus, adhesion, and migration terms was consistent with the wound-closure phenotype. Together, these changes define a coherent transcriptional program that parallels the improvement in wound closure observed following GA-017 co-treatment. However, because our RNA-seq design did not include a GA-017-only group, we cannot determine whether the opposite-direction changes reflect reversal of the Lat-A-associated response or an independent transcriptional effect of GA-017.

Ccn1 and Ccn2 are credible components of that program. RNA-seq and RT-qPCR showed lower Ccn1 and Ccn2 transcript levels after Lat-A treatment and higher levels in the Lat-A plus GA-017 condition. Fluorescence measurements separately showed lower CCN1 and CCN2 signals after Lat-A exposure. Previous studies have shown that CCN proteins can modify cell-matrix interactions and migration, properties that are directly relevant to mesenchymal remodeling. Developmental evidence gives the two genes complementary relevance: Ccn1 is required for embryonic vascular integrity (Katsube et al., 2009; Mo et al., 2002), whereas loss of Ccn2 delays palatal elevation and alters Rac1- and RhoA-associated signaling (Parada et al., 2013; Tarr et al., 2018). CCN1 and CCN2 have been linked to YAP/TAZ signaling in other tissues (Quan et al., 2014; Moon et al., 2020; Lee et al., 2019). Their behavior here is compatible with CCN-mediated changes in the adhesive and migratory state of palatal mesenchyme. However, gene-specific knockdown and rescue experiments are needed to determine whether either factor mediates the GA-017-associated recovery.

Analysis of public single-cell and spatial transcriptomic datasets provided developmental context for the elevating palate. Sample/library-level RNA analysis suggested stage-associated increases in YAP/TEAD-target and migration programs during E13.5-E14.0, and E13.5 spatial sections showed overlapping actin- and YAP/TEAD-related programs. Correlated accessible peaks containing TEAD motifs were identified near Ccn1 and Ccn2, although motif occurrence does not demonstrate TEAD binding or enhancer activity. Interestingly, the lower actin-isoform expression at E14.5 than at E13.5 (Supplementary Figure S7) and the divergence between later actin-transcript patterns and regulatory accessibility also raise the possibility that actin and YAP/TEAD-related programs may become partly decoupled after E14.0, suggesting that actin regulation can be a YAP/TEAD-independent phenomenon at this developmental stage, while TEAD1/TEAD4 occupancy mapping and perturbation of linked elements would be required to test direct cis-regulation.

This study has several limitations. Lat-A and GA-017 are pharmacological perturbations with effects that are not restricted to actin-dependent migration or YAP alone. While these tools provide valuable mechanistic insights, they cannot fully recapitulate genetic loss- or gain-of-function conditions. Moreover, apoptosis and tissue viability were not directly assessed in the organ cultures, and tissue integrity was not quantitatively evaluated. Consequently, cellular injury or altered survival cannot be excluded as contributors to the abnormal shelf morphology, and the organ-culture findings do not establish a migration-specific or YAP/TAZ-dependent mechanism. In addition, direct rescue of palatal shelf elevation by GA-017 in organ culture was not performed. Whether pharmacological YAP activation can reverse the elevation defect at the tissue level remains an important question for future studies. Furthermore, the sample size for stage-level public-data comparisons was small, and the spatial analyses were correlative. Within these boundaries, the data support an association between actin disruption, LATS-sensitive YAP/TAZ-responsive transcription, and reduced palatal mesenchymal wound closure, with CCN1 and CCN2 as candidate mediators (Figure 5U).

## Data Availability

The RNA‐sequencing data generated in this study have been deposited in the Gene Expression Omnibus under accession GSE343005. Public datasets analyzed here are available from GEO under accession GSE218576 and from the Mouse Organogenesis Spatiotemporal Transcriptomic Atlas (MOSTA).
