# Supplementary material for: Actin disruption is associated with altered YAP/TAZ-responsive transcription and reduced migration during palatal shelf elevation
Source: Front Cell Dev Biol. 2026 Oct 1;14:1962374. doi: 10.3389/fcell.2026.1962374 (PMC13668852; doi:10.3389/fcell.2026.1962374)
Supplement: Supplementary file 1 [file DataSheet1.docx]

**SUPPLEMENTARY METHODS**

**GSE218576 single-cell multiome analysis**

GEO-supplied filtered 10x Genomics feature-barcode matrices were analyzed for E12.5, E13.5, E14.0, and E14.5 samples. Palatal mesenchyme-like cells were identified using mesenchymal marker programs, and each public sample/library was treated as the biological aggregation unit. RNA counts were summed within each sample/library and converted to log1p counts per million. Prespecified modules were YAP/TEAD targets (Ccn1, Ccn2, Ankrd1, Amotl2, Areg, Birc5), YAP/TAZ/TEAD core (Yap1, Wwtr1, Tead1, Tead2, Tead3, and Tead4), actin regulation (Actb, Actg1, Actn1, Actn4, Cfl1, Cfl2, Arpc1b, Arpc2, Arpc3, Arpc4, Wasf2, Cdc42, Rac1, Rhoa, Rock1, Rock2, Myl9, Tagln, Flna), and migration/adhesion (Fn1, Vim, Itga5, Itgb1, Mmp2, Mmp14, *Rac1, Cdc42*). The E13.5-versus-E14.0 comparisons used two-sided Welch tests at the sample/library level. For targeted peak-to-gene analysis, peaks within ±500 kb of selected transcription start sites were tested separately in each sample using correlations between log-normalized gene expression and accessibility in palatal mesenchyme-like cells. At least 200 cells per sample were required; genes and peaks detected in fewer than 20 cells were excluded. Sample-level effects were combined and adjusted by the Benjamini-Hochberg method, with positive links defined at FDR < 0.05. Mouse mm10 peak sequences were scanned against selected JASPAR motifs, and motif occurrence was defined by a maximum relative position-weight-matrix score ≥0.85. TEAD-motif accessibility was summarized per sample/library. This targeted procedure tests motif occurrence in expression-correlated accessible peaks; it is not a TEAD occupancy assay, chromVAR analysis, or footprinting analysis.

**MOSTA spatial analysis**

Four E13.5 E1 sections were analyzed using supplied normalized expression matrices, spatial coordinates, and anatomical annotations. The primary oral field comprised bins annotated as Jaw and tooth, Mucosal epithelium, Connective tissue, or Cartilage primordium and was refined using a craniofacial mesenchyme marker program (*Msx1, Prrx1, Prrx2, Shox2, Barx1, Runx2, Dlx5, Dlx6*). YAP/TEAD-target, YAP/TAZ/TEAD-core, actin-regulation, and migration/adhesion scores were calculated as the mean expression of matched genes. Each score was smoothed over the 25 nearest spatial neighbors. Spearman correlations were computed separately within each section and within the marker-refined candidate region. Spatial bins were not treated as biological replicates, and the four sections share E1 provenance; correlations were therefore interpreted descriptively rather than as independent-replicate inference.

**Figure S1. β-catenin staining in posterior palatal shelves during elevation.** β-catenin signal is shown in green and nuclei in blue. Yellow and white boxes indicate the higher- and lower-cell-density mesenchymal regions, respectively. Scale bar, 200 µm.

**A**

**B**

**Figure S2. Isolation and characterization of MEMP cells. (A)** Bright-field image of spindle-shaped cultured cells. **(B)** RT-qPCR analysis of Cdh1 (E-cadherin) expression. Data are mean ± SD. **P < 0.01. Scale bar, 200 µm.

**Figure S3. MEMP cell morphology and F-actin organization after Lat-A treatment.** **(A)** F-actin disassembly across 0.1-2.0 µM Lat-A; DMSO was the vehicle control. **(B)** Recovery after 1 h of 1 µM Lat-A followed by a 24-h washout. In each pair, the upper image is bright-field and the lower image is fluorescence; Control was the DMSO vehicle control. F-actin is pseudocolored red. Scale bar, 50 µm.

**Figure S4. Cellular reducing activity and wound closure during mitomycin treatment.** **(A)** CCK-8 signals after Lat-A exposure. **(B)** At 1.5 μM mitomycin C, the CCK-8 signal showed no apparent increase between 12 and 24 h in MEMP cells. **(C)** Representative wound fields during concurrent 1.5 µM mitomycin and 1 µM Lat-A treatment and quantitative wound-area closure analysis showing significantly reduced wound closure in the Lat-A plus mitomycin group at 24 h. Data are mean ± SD where quantified. *P < 0.05. Scale bar, 250 µm.

**Figure S5. Cell-density-associated YAP localization in MEMP cells.** **(A)** Representative YAP (green) and DAPI (blue) images at low, medium, and high density. **(B)** Cytoplasmic-to-nuclear YAP fluorescence ratios for 10 segmented cells randomly selected from 3 biological replicates per condition (one-way ANOVA, P = 0.00174). Data are mean ± SEM. Scale bar, 50 µm. **P < 0.01. ns, not significant.

**Figure S6. YAP localization after cytochalasin D-mediated F-actin disruption. (A)** Representative YAP (green), F-actin (red), and DAPI (blue) images in DMSO- and cytochalasin D-treated MEMP cells. **(B)** Cytoplasmic-to-nuclear YAP fluorescence ratios for 5 fields of view from 5 biological replicates per condition (Welch ANOVA, P = 0.000563). Cytochalasin D treatment significantly redistributes YAP away from the nucleus. Data are mean ± SD. Scale bar, 10 µm. **P < 0.01. ns, not significant.

**Figure S7. Actin isoform expression during palatal elevation.** (A) Lat-A was associated with lower expression of the tested actin isoform transcripts except *Actc1* in MEMP cells. (B) ACTA2 and ACTG1 staining was higher on the medial side of the palatal mesenchyme during elevation. (C) RT-qPCR showed lower *Acta1, Actb, Actc1, and Actg1* expression at E14.5 than at E13.5. The later decrease contrasts with the E14.5 increase in Ccn-linked TEAD-motif-containing accessibility in Figure 6C, suggesting possible stage-dependent decoupling. White arrows in B indicate increased ACTA2 and ACTG1 staining of medial palatal shelves at E14.0. Data are mean ± SD. Scale bar, 50 µm; *P < 0.05, ***P < 0.001, ns, not significant.

Table S2. Primer sequences used for RT-qPCR.

| **Gene** | **Sequence** |
| --- | --- |
| *Acta1* | F: 5′-TCCAGCCTTCCTTTATCGGTATG-3′  R: 5′-CTGACATGACGTTGTTGGCATAC-3′ |
| *Acta2* | F: 5′-CCAGCTATGTGTGAAGAGGAAGA-3′  R: 5′-TTGGTGATGATGCCGTGTTCTAT-3′ |
| *Actb* | F: 5′-ACTGTCGAGTCGCGTCC-3′  R: 5′-CTGACCCATTCCCACCATCA-3′ |
| *Actc1* | F: 5′-GGAGAAGATCTGGCACCATACAT-3′  R: 5′-ATGAGTTACACCATCGCCAGAAT-3′ |
| *Actg1* | F: 5′-CCGATCGCAATGGAAGAAGAAAT-3′  R: 5′-GTCACCCACGTATGAGTCTTTCT-3′ |
| *Actg2* | F: 5′-GGGATCTCACAGACTACCTCATG-3′  R: 5′-ATTCTCGAAATCCAGGGCTACAT-3′ |
| *Gapdh* | F: 5′-CAGTGGCAAAGTGGAGATTGTTG-3′  R: 5′-TCGCTCCTGGAAGATGGTGAT-3′ |
| *Cdh1* | F: 5′-GGTCATCAGTGTGCTCACCTCT-3′  R: 5′-GCTGTTGTGCTCAAGCCTTCAC-3′ |
| *Ccn1* | F: 5′-GTGAAGTGCGTCCTTGTGGACA-3′  R: 5′-CTTGACACTGGAGCATCCTGCA-3′ |
| *Ccn2* | F: 5′-TGCGAAGCTGACCTGGAGGAAA-3′  R: 5′-CCGCAGAACTTAGCCCTGTATG-3′ |
